# Adult postgraduate students’ learning in online spaces—Images of identity and belonging

**DOI:** 10.1007/s40955-021-00187-2

**Published:** 2021-10-01

**Authors:** Carol Azumah Dennis, Gill Clifton

**Affiliations:** The Open University, Milton Keynes, UK

**Keywords:** Adult postgraduates, Online learning, Image-based methodologies, Portrait methodology, Retention, Erwachsene postgraduierte Lerner, Online-Lehre, Bildbasierte Methode, Porträt-Methode, Retention

## Abstract

This research seeks to animate the voices of postgraduate students registered on a UK distance learning online Masters in Education or Childhood & Youth programme. Such a critical exploration is timely given the HE landscape is premised on its openness and accessibility. Our study reports on 33 interviews with postgraduate students using photo elicitation and unstructured interviews. We prioritise the perspectives of students whose experiences do not replicate the success stories which generally epitomize representations of HE study, favouring instead the voices of students who interrupted or in some cases terminated their studies. Our aim is to better understand the PG students’ personal, professional, and academic learning trajectories.

In reading the data we produced four “manifesto” statements crafted from a series of dialogues between ourselves as researchers, our colleagues, the online experiences of adult postgraduate students and our reading of literatures surrounding withdrawal, persistence and retention. Interpretations gravitated towards four themes: identity, belonging, digital pedagogies and uncanny spaces and which point towards students’ perspectives about the interconnections between identity and belonging and how these concepts help develop understanding of “social presence”, what Bayne (2008) and Cartens (2016) assert as “uncanny” spaces. Our manifesto statements represent our reading of the data to stimulate further thinking around the HE digital pedagogy landscapes. The four statements have implications for how we understand, participate in and manage postgraduate adult students’ learning in digital spaces.

## Abstract (de)

Dieser Artikel gibt Studierenden eines britischen Online-Masterstudiengangs in „Education“ eine Stimme. Eine solche Untersuchung scheint im Rückgriff auf den Anspruch der Offenheit und Zugänglichkeit des britischen Hochschulsystems zeitgemäß. Die Untersuchung basiert auf 33 Interviews mit Studierenden eines Postgraduierten-Programms, die in unstrukturierten Interviews befragt und begleitenden Fotografien festgehalten wurden. Wir nehmen dabei die Perspektive der Studierenden ein, die nicht die üblichen Erfolgsgeschichten des Hochschulsystems wiedergeben, sondern favorisieren stattdessen Stimmen von Studierenden, die das Studium unter- oder abgebrochen haben. Ziel der Untersuchung ist es, die persönlichen, beruflichen und akademischen Ziele der Studierenden zu rekonstruieren und zu verstehen.

Bei der Auswertung der Daten wurden vier Aussagen herausgearbeitet, die sich aus Gesprächen mit Forschenden, mit Kolleginnen und Kollegen, aus Erfahrungen in der Online-Lehre sowie aus der Auswertung von Sekundärliteratur ableiten ließen. Die Interpretationen kreisen um Themen wie Identität, Zugehörigkeit, digitale Pädagogiken und „unheimliche“ Räume und ermöglichen Rückschlüsse auf deren Verwobenheit untereinander. Dies wiederum ermöglicht ein Verständnis des Konzepts „sozialer Anwesenheit“, die als „unheimlich“ charakterisiert worden ist (Bayne 2018; Cartens 2016). Hiermit wird ein Beitrag zum Verstehen geleistet, wie Studierende in digitalen Räumen agieren und lernen.

## Introduction

The extraordinary events of 2020 pivoted post-16 teaching, learning and research into a series of discontinuities. COVID-19 and the imposition of social distancing and lockdown did not precipitate the shift to online pedagogy for Higher Education in the UK, that shift had been underway for some time (Rospigliosi 2020). But it did exaggerate and accelerate it. It also placed the issue, digital pedagogies, on a public agenda. Most often the public discourse that has surrounded socially distanced pedagogies is one of disquiet. It is driven by the commodification of HE and the assumption that in the absence of an encounter surrounded by a bricks-and-mortar lecture hall, students are getting an inferior product and are therefore deserving of a refund on their £ 9000 per year student fees. These discussions have placed emphasis on the undergraduate, HE population, our focus in this paper is on postgraduate students.

Our research participants were adults who, having returned to study often after a long break, interrupted or terminated their programme. This is quite a distinct population. Much of the research into withdrawal is concerned with full-time, 18- to 21-year old undergraduates. Studies tend to explore factors that influence undergraduate students’ capacity to remain on course and complete their degree, frequently citing as highly significant things such as family background, being homesick and developing friendship groups (Wilcox et al. 2005). There are some studies that focus on adult students and doctoral level students (Keefer 2015). The lacuna seems to surround taught postgraduate, master’s students who are adults studying part time (Schroeder 2019). In her unpublished doctoral thesis Schroeder (2019) remarks on the low number of usable studies returned when conducting a search using the key terms “Master Student Persistence,” “Master Student Retention,” and “Master Student Progression.” In so doing she echoes a gap identified by Mercer (2015) four years earlier in 2015 and Gordon (2016) a year later. Our literature search in 2020, using identified key phrases and a university data base, found a field that has so far remained unanimated. The specific context of online learning merely exaggerates the paucity of literature. Based on institutional data, the cohort who participated in this study—postgraduate students at a distance learning university—come from varied backgrounds and locations. They are typically older than their counterparts in face-to-face universities. While the MA requires that students have completed an undergraduate degree, entry to the undergraduate programme requires no prior qualifications. Those students who progress onto the MA from an undergraduate degree in the institution thus have an atypical academic background. Many of the students are working full or part-time, indeed, the flexible nature of anytime, anyplace distance learning makes it attractive to working students. There are a higher than usual number of students with additional needs and/or disabilities in comparison to other UK universities (The Open University 2018). Caught between undergraduate education, in which some 50 % of the UK population participate, and high-status doctoral studies, the MA is the empirically overlooked middle child of UK Higher Education. The emotional experience of distance learning students has also received little focussed attention when compared to other student groups. Given the particularities of the cohort we work with, one can assume that their internal emotional lives will be equally as distinct (Hilliard et al. 2019). This is our current focus. We animate the space surrounding the experiences, challenges and opportunities for adult postgraduate (masters) students engaging with online learning.

## Project “50 narratives @ 50”—Portraits of persistence, progression and belonging

The project we recount in this paper, “50 Narratives @ 50” (Dennis 2020), used a milestone in the life of an established distance learning University in the UK to interview adult postgraduate students using photo elicitation and unstructured interviews. Here, we report on the 33 interviews conducted between September 2019 and June 2020. Our exploration prioritized the perspective of students whose experiences of study did not replicate the smiling success stories which epitomize publicity friendly representations of HE. Our primary purpose was to investigate students’ lived experiences following a period of postgraduate study that had been interrupted or in some cases terminated, to understand students’ personal, professional, and academic learning trajectories. The research was of vital importance to our ongoing critical exploration of the openness of a Higher Education sector that prides itself on being “open”. While we appreciate the importance of retention and key performance indicators for HE, the research did not set out to understand reasons for non-completion but rather the narratives that surround withdrawn students and their perspective on if and how their learning episode has value.

After obtaining a favourable opinion from the University ethics committee, we sent 600 email invites to students who had at some point registered for the fully online Masters in Education, Childhood or Youth Studies and either interrupted or terminated their studies. There were two distinct parts of the data generation. First students were invited to provide an image which represented their experiences of Post Graduate study. While we anticipated that research participants would generate their own images via photographs they had taken, the majority of images presented were found images. Nonetheless, whether found or self-generated, the images allowed students to talk about their experiences in a way that language alone might have hidden. They generated intensely vivid, personal, unexpected and sometimes absurd metaphors which allowed us to view the PG study experience in novel ways. Once we had secured an image, we conducted a one-to-one video interview via Microsoft Teams. The images did not speak for themselves. Our first question was to invite research participants to discuss the image—to describe it and explain what they felt it conveyed. This was followed by an open unstructured interview with a defined set of guidelines for what the interview would include but no specific questions and no structured order to follow. The aim was to create conversations negotiated with as much fluidity as possible. Working within an open protocol our interest was in the generation of narratives. This study shares with all forms of narrative enquiry, a common interest in the study of experiences (Clandinin and Rosiek 2006). However, while we do have an interest in themes, we also have a strong impulse to listen to individual voices. We wanted holistic narratives, combining holistic-content and categorical-content (Leiblich et al. 1998). We also placed emphasis on a dynamic retelling of experience. We wanted to know what happened and what happened next. What did they do, think or feel and why? Who was involved in their story? During the interviews prompts were focussed around—what happened next placing each account in a specific bounded context of time and ongoing action. We were mindful in this process, of placing little emphasis on direct interrogation of motivations or interpretations of postgraduate study. Moerman’s (1974) seminal advice about the limited value of questions which generate abstract accounts provided at the behest of an ethnographer, meant that throughout the interview we avoided asking—what’s it like doing and failing to complete postgraduate masters study with a distance learning university preferring when-, how-, and why-questions: time and action. The narrative form adopted is one that allowed for the recounting of events, recollection of experiences and relating emotion. In collecting and attempting to understand what these individual research participants tell us, we connect the intricate intimacy of a personal life to larger situational, organisational, cultural, and historical narratives.

## From narratives to narrative portraits

Interviews were conducted in Microsoft Teams, the preferred communicative platform for the University also consistent with the social distancing conditions created by COVID-19. Once recorded, the images were filed, and interviews transcribed verbatim. Each transcribed interview was then crafted into a narrative portrait.

Portraiture is a method of qualitative research that blurs the boundaries of aesthetics and empiricism in an effort to capture the complexity, dynamics, and subtlety of human experience. In portrait methodology rigor, subjectivity, aesthetics, and art (broadly defined) are complementary ideals (Lawrence-Lightfoot and Davis 1997). In our approach which we viewed as listening *for* a story as more important than as listening *to* a story. In developing narrative portraits we explored each transcript to identify an initiating event, a pivotal moment and something of a resolution. The words used to craft the portrait are drawn exclusively from the interviews with the role of researcher being to identify the relevant narrative moments which are woven into a whole. The stories told are differently each time and they are the story of the narrative subject rather than their registration, experience on and withdrawal from their masters. These narrative portraits are crafted by selecting and rearranging extracts from interviews to offer a glimpse into the subjects’ lives (Rodríguez-Dorans and Jacobs 2020) an approach associated with narrative inquiry (McAdams 2008), narrative profiles (Seidman 2006). We identify these as narrative portraits rather than merely portraits as they are based on narratives. If a portrait emphasises space, shape, contour and context, narratives pivot around time and action. To understand the experience of postgraduate (masters) students in an online university requires not only a profile of who they are but a holistic account of what happened to and around them, what they did and why.

Having generated our images and portraits, we were keen to avoid a quasi-positivistic hankering that often betrays the interpretivist’s treatment of data: coding, abstracting and categorizing into visual and narrative themes. We chose to remain faithful to the artistry involved in what are after all acts of interpretation. We do not subscribe to an epistemology based on a singular plot, underlying causal connections that determine our social world or concrete immutable patterns discernible only through authoritative interpretations (Usher 2001). Therefore, instead of subjecting the data to continual rounds of computation, parsing, subsummation and analysis—we adopted a process *reminiscent of an analytical daydream*. We read and re-read the data, meeting on a bi-weekly basis to discuss our different interpretations of what we found, reverberations within and between what we had generated, what we were reading and our wider experiences of postgraduate (masters) study. We equivocated between theory and data, sometimes reading up from data to theme, other times reading down, recalling what others have written (MacLure 2008), listening for echoes and resonances. We do not present a traditional—data, findings, conclusion—account of our research. Working within an interpretative tradition, the truths that have emerged through the richness of participants’ images, narratives and portraits provide ethnographic insight that reach towards fuzzy generalisations (Bassey 2003).

The data did not speak for itself, nor did it speak with one voice nor follow a singular narrative thread. We encountered an emerging undulating geography. There were cul-de-sacs and roundabouts, ideas that felt important and interesting but had only a shallow resonance. There were also strands of thought and experience that refused to be distorted into clarity (Law 2004). In reading the data we resisted the impulse towards a naturalistic linear tale.

We have also resisted the compulsion to present individual “cases” comprising of biographical narratives. Instead, having generated the data, crafted narrative portraits and listened to what research participants have said about the images they chose to present, we offer four “manifesto” statements. Manifesto in the sense that written at a moment of transformation, they make visible our sense of what stepping forward into and through the digital transformations of adult postgraduate study might mean (Latour 2010). The statements were crafted from a series of dialogues between ourselves as researchers, our colleagues, the online experiences of adult postgraduate students and our reading of literatures surrounding withdrawal, persistence and retention. The four statements gravitate around four themes of identity, belonging, digital pedagogies, and uncanny spaces.

### Statement 1: Adult learners like nomads traverse multiple identities, some they actively seek out; others are driven by a sense of need or obligation

(Fig. 1).

**Fig. 1:**
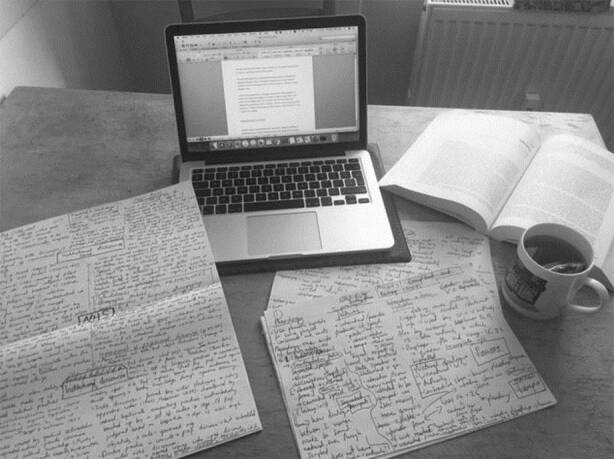
Image provided by Student Interview CEd #2

Students encounter multiple identities, some they pass through fleetingly and over time, others they settle into establishing permanent, all-consuming and long-lasting roots. Some identities are imposed by political, economic, and social structures that frame contexts and experiences; others are entered into willingly, actively seeking them out in order to “belong” (Abes and Kasch 2007; Thomas 2018). The act of seeking an identity which enables one to belong can be consciously driven by need, obligation, expectation or unconsciously engineered by societal influences and political, economic structures (Lambert et al. 2013). Belonging is central to understanding how students experience higher education. What, how, where and the extent to which research participants identified with their student identity being inextricably linked to the extent to which, how and to what they feel they belong. These concerns are frequently overlooked in postgraduate students. The assumption seems to be that having already experienced success as an undergraduate, identity and belonging are no longer pertinent to the postgraduate experience and do not need empirical exploration (Tobbell and O’Donnell 2013).

In this section we consider the tensions and challenges of multiple identities which arose from the struggles students encountered while navigating their different roles. They brought into sharp relief the embodied nature of online learning, especially when students are confined to studying within a tightly constrained space. It should be noted that learning at a distance and in the case of participants in this research, fully online, poses unique challenges for students (Tareen and Haand 2020). Online learning providers often assume postgraduate students know what to expect from online learning; they assume students have knowingly and actively chosen this mode of study to fit in and around their specific circumstances. Those who may have attended bricks-and-mortar HE Institutions will draw on the resource of previously embraced successful student identities; those who have not before experienced HE study online will have fewer and less clearly defined expectations. Either way students are entering an unfamiliar environment and inevitably bring perceptions of what it might look and feel like. But the absence of a lecture hall, library and campus allowing students to physically remove themselves from one space and enter another clearly defined study space creates tensions:

What I hinted at with the [image provided] was this negotiation of space and the relationship, […] I had a lot of arguments [with my partner] about […] my availability and about my tendency to duck out of life when a deadline was approaching and not being available.

Student Interview CEd #2

Student interview CEd#2 reveals a complex personal and professional identity which involved the negotiation of physical space. She chose to undertake PG study hoping to develop a deeper appreciation of her job, a key motivator for postgraduate students (Tomlinson and Jackson 2019) especially in Education. She was struggling with the challenges of fitting in study time but feeling that it was also an outlet from the pressures and demands of work. In her narrative she highlights the not unfamiliar tension in many households in respect to coming to terms with her identity as a student which meant she was unable to take full ownership of her identity as a supportive partner to someone who was also studying. The outcome for this research participant was

[…] constantly feeling torn in different directions and guilty.

Student Interview CEd #2

CEd#2’s narrative has strong echoes of Illeris’s (2014) theorisation which points towards the importance of recognising learning, as well as transformative learning which requires more than a somewhat superficial change in meaning perspectives, frames of reference and habits of mind (Mezirow 2006). The inclusion of social and emotional dimensions of learning only avoids being too narrowly focused on cognition when the various elements are bought together in the concept of identity. Our suggestion is that CEd#2 makes an incomplete transformation in her student identity and was thus unable to experience and embody academic belonging to the exclusion of other calls on her space, time and attention. We see in these similarities to Bayne and Jandrić (2017) notion of doubleganger, the double of a living person who inhabits the online digital world of second life. Might it be that research participants like CEd#7 experience multiple versions of themselves? This is a discomfort associated with online interaction, a discomfort Bayne and Jandrić (2017) links intimately to inhabiting “uncanny spaces”. The unease experienced in online learning is qualitatively distinct from that experienced in face to face encounters. The eeriness of a disembodied presence paralleled by an embodied absence (Hook 2005) in the context of the immediacy of digital spaces, means students have little time to feel their way through. The online world has an immediacy not mirrored in the physical world. It can be brutal, unforgiving and demanding, silencing those who feel they are invading the space of others rather than believing they belong and have validity in that space.

I was going to [complete the module] but then I got ill. I was ill for about two years and then I’m where I am just now. When I go back to the [university], and I think I will go back to the [university], I’m going to go back to do something that is probably not academic in that sense and it’ll be about literature or it’ll be about writing.

Student Interview *CC #3*

Harrison et al. (2003, p. 6) offers a useful analogy of “fuzzy boundaries” commenting about the transitional notion of identity and how identities are often woven together. It is the attempt at weaving incommensurate identities together that creates a collision course—particularly if as Student CC#2 identified, you feel trapped and unable to navigate between competing demands on who you are and where you belong.

I knew that I couldn’t do it, I was completely defeated, I was like, ‘No, I know how much headspace you need to write the last one {assignment} and I haven’t got it in me, I can’t do it,’ and I just gave in, you know?

Student Interview CC #4

### Statement 2: To belong is a fundamental need: it is only by owning “belongingness” we can fully embrace a sense of fulfilment that emerges from being with others

(Fig. 2).

**Fig. 2:**
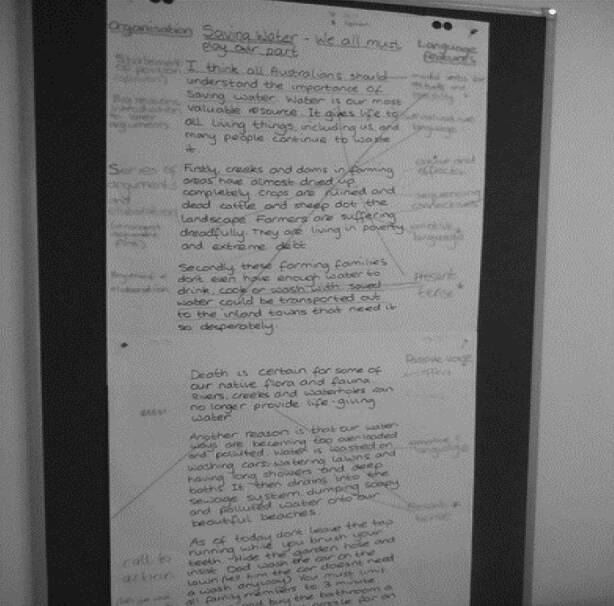
Image provided by Student Interview CEL

To belong is a fundamental need (Strayhorn 2018). Feeling at ease in pedagogic spaces makes a decisive contribution towards educational success. It implies more than just being present. Belonging comes from knowing you have a right and a purpose to physically and abstractly be where you are; when you belong and call a place “home” you assume the right to be in control, giving yourself the right to dip in and dip out, leave and return at will. These narrative portraits were of students who had withdrawn from their programme. We inevitably encountered stories that had an emotional impact, stories of failure, frustration and dreams deferred.

I took my degree when I was 50, decided to have a total career change and went from basically a childminder to a qualified teacher in eight years, that is my learning history. So, you know my learning history is very different to someone who is just graduating through university. I […] always thought myself capable and able to learn, loved the whole learning thing, very enthusiastic about learning. And then this was like somebody just, if you are riding a horse and it pulls back on the reins really sharp and the horse came to a dead stop, but at the same time sat back on its haunches if you like, so it literally made me sit back onto my haunches and think, ‘I can’t do this,’ […] I had lost my confidence totally about learning after that experience. […] And I don’t give up easily […] I love learning, but it was beyond me, it was just too much.

Student Interview CC #6

Narratives most often included an element of belonging. Sometimes the process of learning and of belonging to the very act of learning; other times Higher Education as an institution was considered a “home” to which students wanted to return even though there may have been an interruption to their study, they had started on a journey and they would return. This was a remarkable feature of the narratives given these research participants had all withdrawn from a master’s programme. It was not what we were anticipating.

I […] went on and did […] a couple of Level 3/Level 4 qualifications in totally unrelated topics and did that online and did fine.

Student Interview CC #6

I’ve got the craving to learn […] I still want to learn and, you know. I think if I was lucky enough to have another opportunity to study with […] University, I’d like to do an open masters.

CC #4

We suggest that the significance of these narratives is not that they feel warmly towards a particular institution, but that they evidence a sense of belonging. There is a body of literature that evidence belonging as an important aspect of student retention (O’Keeffe 2013). A strong sense of belonging sits at the heart of student retention (Thomas 2012). A less well explored thread is the role of belonging amongst students who withdraw from postgraduate study. Research participants’ narratives suggest they retain a sense of belonging and a desire to return to postgraduate study. This was not a sentiment we were expecting to find. This arguably reframes the concept of student retention, placing it in a completely different narrative time frame. Student success, bounded in programme terms, is charted from the moment of registration to the moment of certification or withdrawal. These narratives suggest a frame that slips beyond that defined by the institutional programme from learning desire to learning fulfilment, which may involve registration, withdrawal and either re-registration or the fulfilment of learning desire in unexpected, unanticipated and unpredictable ways. We suggest that it is home, a sense of belonging that make students feel they have a place (which in some instances is an institution) to which they can return.

With the [university] though, I’ve heard so many amazing stories about it, especially working with PhD students, those who did their undergrad with the [university], did their masters with the [university], now doing their PhD with the [university]; they, you know, consider it like part of their family pretty much, because it’s been such a large part of their life for years and years.

Student Interview CEL #1

The home we are suggesting here might be an institutional home, but might also be a disciplinary home, it might even be something completely intangible, a notion of a process or community as home: something to which they will return at some stage.

You know, I love learning so much […] I really want to study again, I really, really want to do a masters, and I’m really fed up because I shelved it. In my mind I shelved it. And I’ve come out of it with a […] You know, I owe [the student loan company] money for it, you know, and I’ve got this debt for this module that is incomplete.

Student Interview CC #4

### Statement 3: Whilst working at a physical distance it is possible to create safe and enduring environments in which digital pedagogies flourish and learning prospers

(Fig. 3).

**Fig. 3:**
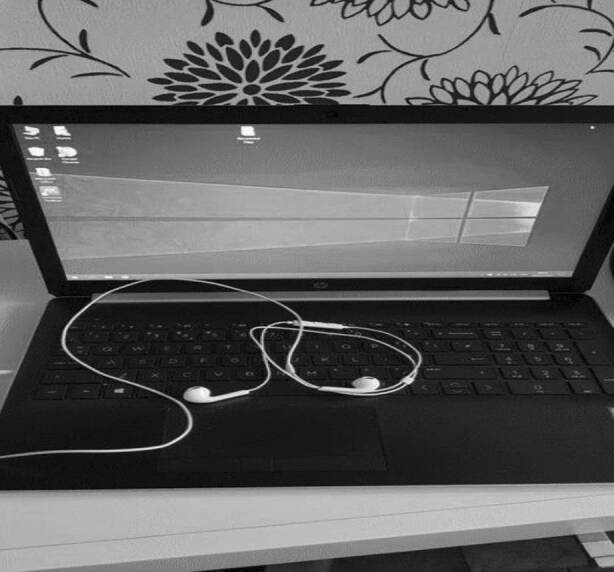
Image provided by Student Interview CC #7

I think one of the other benefits of the digital university study is the lifeline it can give you to feel connected to an academic community and an academic process, even when you are living and working in a context where you don’t have access to that.

Student Interview CEd #2

The press, public and politicians view of online pedagogies, pivot on the belief that *online learning* is something other than *learning*. If students are not removed from their daily living space to attend a physical campus building and sit in a large hall, they are receiving an inferior product. While much of this public discourse is filtered through the specific commodification of UK higher education (Silverio et al. 2020) the notion that online learning is something other than learning is an accepted proposition.

In this section we consider this disquiet—or rather allow the data we have generated to disrupt it. It seems to rest on a misconception. A misconception that online learning in some fundamental way takes us out of and away from ourselves, perhaps even dehumanizing us. It is as if distance disconnects us: a person mediated by a computer screen is less of a person. The anxiety experienced here is reminiscent of that envisioned by Isaac Asimov in his 1950 sci-fi novel, The Naked Sun. The Solarians (inhabitants of the planet Solaria) interact with each other entirely through holography and screen. Digital mediation, it seems, renders us—and our relationships including our pedagogic relationships, less authentic. Digital mediation transforms us into aliens.

#### The teacher hologram

The image most frequently posted by research participants was that of a computer screen and keyboard. Given the context of a distance learning university, this is perhaps obvious to the point of cliché. But it also points towards the possibility of disruption. In the absence of a bricks-and-mortar lecture hall, the ontological status of the pedagogic space—the seminar room or the lecture hall—is called into question. If teaching is thrown into doubtful disarray, the other side of the pedagogic equation, learning can hardly remain intact. The images of computers took several forms—sometimes they were artfully arranged with coffee cups, framed by reading material and handwritten annotated scripts. Other times they were part of a creative ensemble with other objects—plants, pets, knitting and glasses. In some instances, they included images of students writing either alone or in company (children in the background).

The default mode of higher education is face-to-face lectures though of course pedagogies are changing. The postgraduate (masters) study these research participants experienced was entirely online. For many students this was an unusual experience. And while many of the research participants had completed an online undergraduate degree, for others the masters was their first experience of online learning.

Student CEd#7 describes encountering online learning for the first time.

It’s a completely different experience because obviously, [in a bricks-and-mortar University] you’re interacting with people, you’re going to lectures in theatre rooms and tutorials with your professors and so on and so forth—and so, sitting at home at your desk or at your dining table, or any other space that you can find in your household to do stuff was a new experience.

Student Interview CEd #7

This student, CC#7 presented an image of her laptop. Nothing else is visible except the edges of a desk and the hint of an ornately wallpapered wall. A pair of headphones are strewn across the keyboard. She explains the significance of her image

Yeah … the laptop: it’s definitely part of my study journey. It allowed me to, obviously, do assignments, take part in various tutorials; it gave me access to tools like—one of the members on the course opened my mind up to a […] fantastic tool. […] Yeah, so the laptop signifies everything about being a distance learner, really, and being able to participate.

Student Interview CC #7

As her account makes clear, it is human contact she values “interacting with people” or the inspiration of being introduced to a new tool by a colleague, and “being able to participate” all made possible by the computer. The image of a computer and the discussion of study spaces it instigates serve as a reminder that online learning does not exist outside of a physical realm. It is part of a material world of movement, placement and practices. It involves (extends) the body and the person whether they are inspired, bored, lonely or engaged, and is thoroughly entangled with the technology as part of an assemblage. Despite its online nature, there is nothing virtual about CC#7’s learning. Despite having an incomplete MA, there is nothing incomplete about the impact postgraduate study has had on CC#7.

I think, when I started reading the material, it really connected with me because it was a real area that I wanted to find out more about, from a theoretical and academic point of view around, […] the study of young people and youth. A lot of it was really interesting to me on more of a pleasure kind of reading level …

Student Interview CC #7

She talks here about reading for pleasure, but also her growing sense of professionalism. Reading enabled her to understand more fully and more empathetically her pupils; alongside her image of a lap top computer, she uses and explains with pride the concept “adverse childhood experiences” and how this impacts her pupils. The phrase itself is one that resonates deeply for her. It seems to confer both belonging (to a school community and to a profession) and status, the status of someone who makes senior managers sit back and listen.

I […] am much, much, much more informed about my cohort, even more so than some of the very experienced teachers […] at the end-of-day meetings that we have, and I have thrown in […] the term […] ‘adverse childhood experiences’, people like the deputy head and the head […] sit back and listen because, it encompasses everything that our young people are about, […] [the incomplete MA has] brought a wealth of knowledge […] I can then talk with some authority and clarity on young people.

Student Interview CC #7

CC#7’s experience of being taught by the digital university has a profound impact on her sense of self in the workplace. What she learns establishes her professional worth and allows her to talk and be listened to by senior managers. She is driven by a desire to know more about young people and to understand their troubled lives. She has a sense that career progression has become an open possibility. The openness she experiences is not just the openness of an institution designed to make higher level study available to all. It is an openness made possible by her (incomplete) participation in higher level study. This learner has prospered as a result of her partial participation in postgraduate study, for reasons perhaps hinted at by a research participant CC#4.

I could walk in a room or somewhere and nobody knows I’ve got my certificate but if I say something that I’ve learned.

Student Interview CC#4

We are not advocating here a celebration of the fractured postgraduate programme. Whether conceptualised in institutional or individual terms, a complete MA is almost always better than an incomplete one. A sentiment echoed by at least one research participant who included an image of her degree qualification to represent her MA experience. Our suggestion is that the emphasis placed on retention when completing programme reviews is one premised on the needs and interests of the HEIs rather than taking account of the learning desires, purposes and experiences of students.

Not every research participant story was one of unexpected success. But there were pivotal moments when students made a separation between certification and learning. In these moments, it was the learning they valued.

I didn’t finish but […] sometimes you could have the certificate and you don’t really have much knowledge, but I do have the knowledge […] I thought that’s fine because all is not lost.

Student Interview CEL#4

There is a complicated choreography involved in navigating fluid identity spaces. The concept of the “virtual” is replete with notion of non-materiality and disembodiment (Gourlay 2021, p. 57). It encodes an idea of things which exist outside of the physical, material movement, placement or practices, spectral objects which do not involve the body, or the “person”, a binary to “in person”. It is a disembodied realm. The virtual is something other than the real. The real is its corollary. The classroom or lecture hall once unhinged from their bricks-and-mortar moorings, confuse what it means to be a student, requiring reconceptualisation and renegotiation.

There were several moments when we saw in the images and accounts student offered about their experiences, the uncanny, a notion explored by Bayne (2008). The uncanny, associated with the strange, weird and mysterious, a ghostly disembodied presence, accentuates feelings of uncertainty, a feeling that one’s very self is cast into doubt and is questionable.

### Statement 4: Despite their pervasiveness, online spaces can give rise to feelings of disconnection and disorientation, the “support from people who really are interested in what you’re doing” makes a decisive difference

(Fig. 4).

**Fig. 4:**
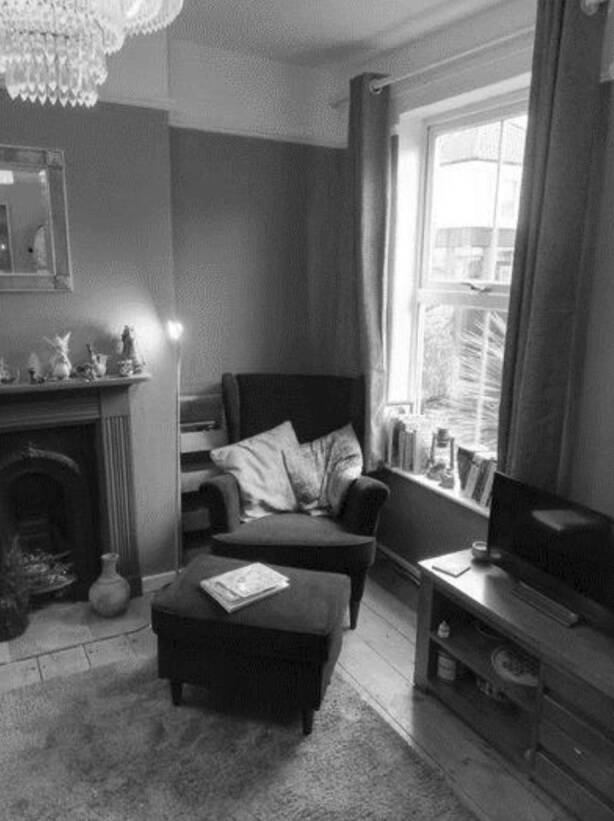
Image provided by Student Interview CC#9

The fluidity of identity and unanchored feelings of belonging are all aspects of uncanny spaces. The uncanny generates disconcerting feelings of strangeness, a sense of the familiar and the strange commingling: the familiar made strange. Conceptualised by Bayne (2008) drawing variously on Royle (2003) the literary and cultural theory of (Kristeva 1991) and Freudian (1919/2003) psychoanalysis, this helped us make sense of some of the more confused narrative portraits. There were moments when students spoke of their ill-ease, discomfort and confusion, which sometimes spilled over into a questioning of self.

I would have given you another image, but I just thought how come I’m the way I am?

And a shifting of identities.

Teachers and learners are making sense of a whole lot of different kinds of information all the time and trying to find a coherence to them and sometimes in those situations you are the learner, sometimes you’re the teacher […] sometimes you’re a Moomin, helping pull somebody out […] you know, struggling in the water and somebody’s trying to help you out, and sometimes your roles change. And sometimes you can be all those things in the space of about three minutes as a practicing teacher or as an ITE lecturer and actually as a learner.

Student Interview CC#3

These discomforts stand at the definitional core of digital pedagogies. The culture of the bricks-and-mortar classroom cannot be replicated through the architecture of the web. The physical infrastructure of bricks-and-mortar is neither the binary opposite or equivalent to the 1 s and 0 s of a digital learning platform (Stommel et al. 2021). While the digital is defined as that which can be reduced to binary (Horst and Miller 2012), the exponential growth in the universality this coding enables stands as corollary rather than opposition to cultural proliferation. While much of the debate about the digital is couched in terms of the threat it poses to teaching, learning and being a student, we are suggesting that genuine academic engagement also happens between keyboard, camera and smartphone in a committed learning community, a redefined classroom.

Online education can be as personal as face-to-face pedagogy. As Horst and Miller (2012) point out as a fundamental tenet of digital anthropology, we are just as human in the digital world and moreover, humanity is not one iota more mediated by the rise of the digital. As Goffman (1974) has repeatedly pointed out, we fail to see the framed nature of face-to-face teaching because the frames work so effectively, they are rendered invisible. The point we are making here is that the decisive difference is connection:

I think one of the other benefits of the digital university study is the lifeline it can give you to feel connected to an academic community and an academic process, even when you are living and working in a context where you don’t have access to that.

Student Interview CEd#2

[…]—it’s been nice to have that support from people who really are interested in what you’re doing. It’s having a bit of—finding out the personality of people and just knowing that you can talk to them about things.

Student Interview CEL#3

## Conclusion

Based on photo-elicitation and narrative portraits crafted from open interviews, we have explored online pedagogy at a pivotal moment of social distancing necessitated by COVID-19. We have amplified the voices of 33 research participants, students who have interrupted or terminated their study while on a postgraduate (masters) programme. The narratives were not as we anticipated. While belonging is frequently cited as a determinate of student retention, these research participants evinced a strong, though perhaps diffused, sense of belonging. Sometimes their entanglement gravitated around an institution, at other times is was more abstract, focussing on a profession, at other times a process. The narratives revealed a yearning for continued study. Learning desire had been neither fulfilled nor negated. In many ways these were incomplete stories of withdrawal and retention, but in another sense they were celebrations of what had been achieved and gained—a story to be continued. The time frame within which they operate was out of sync with the time frame counted by the performative culture of HE accountabilities. Withdrawal from a programme of study followed a distinct parallel track to that of withdrawal from the desire to learn. They have independent start and end points. We are not here beholden to or attempting to generate grounded theories.

The aim of this paper was to do more than reduce and retell data. We experimented with an approach to analysis that involved reading up (from data to theme) and reading down (from dialogue to data). With this we considered identity and belonging, within the context of a wholly online programme in a distance learning university. As a determinate of student retention belonging seemed to exceed its traditional institutional, HE frame. Students interrupted their study but retained a sense of belonging. The notion of the uncanny helped us make sense of the ways in which the digital is somehow experienced as inauthentic and troublesome. Despite this, we drew towards the conclusion that our lives are no more mediated by the digital then by other more established and therefore invisible technologies. Human connection is what makes the pedagogic encounter meaningful. What followed the interrupted MA was variable, what brought our participants together was a continued pedagogic desire and an opening up of life possibilities.
